# Supplementary material for: Single cell sequencing identifies clonally expanded synovial CD4+ TPH cells expressing GPR56 in rheumatoid arthritis
Source: Nat Commun. 2022 Jul 13;13:4046. doi: 10.1038/s41467-022-31519-6 (PMC9279430; doi:10.1038/s41467-022-31519-6)
Supplement: Supplementary file 1 — Supplementary Information [file 41467_2022_31519_MOESM1_ESM.pdf]

Supplementary Figure 1

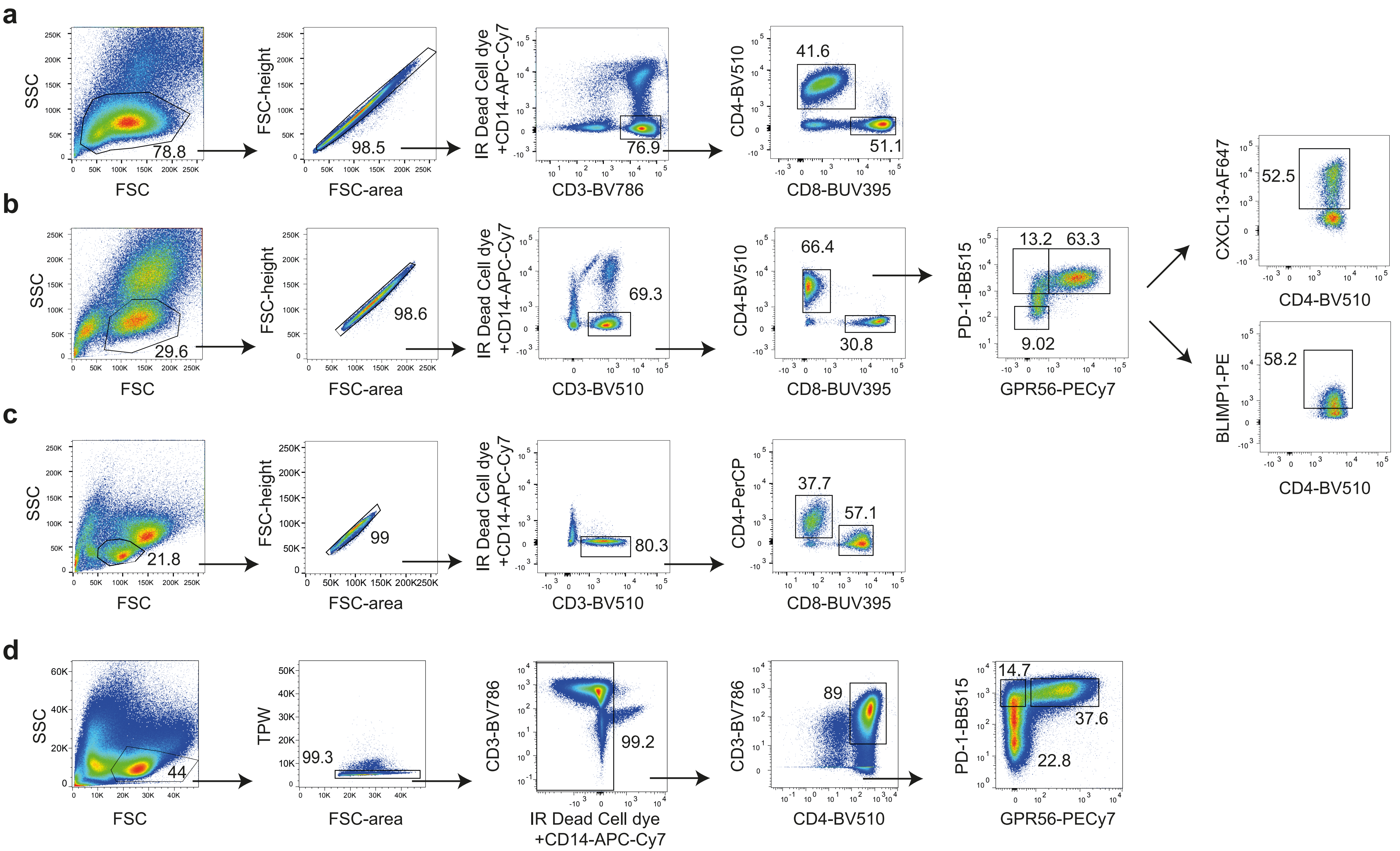

Supplementary Figure 1. Flow cytometry strategy included lymphocyte gating (based on forward and side scatter) followed by doublets, CD14+ and dead cells exclusion. CD4+ or CD8+ T cells were further selected among CD3+ T cells. **a)** Gating strategy for Figure 1 and Supp Fig 2. **b)** Gating strategy for Figure 3 where PD1high GPR56low, PD-1high GPR56+ and PD-1-GPR56- cells were selected. **c)** Gating strategy for Figure 4 and Supp Fig 14. **d)** Gating strategy for flow cytometry sorting of PD-1highGPR56low, PD-1high GPR56+ CD4+ cells for Figure 6.

Supplementary Figure 2

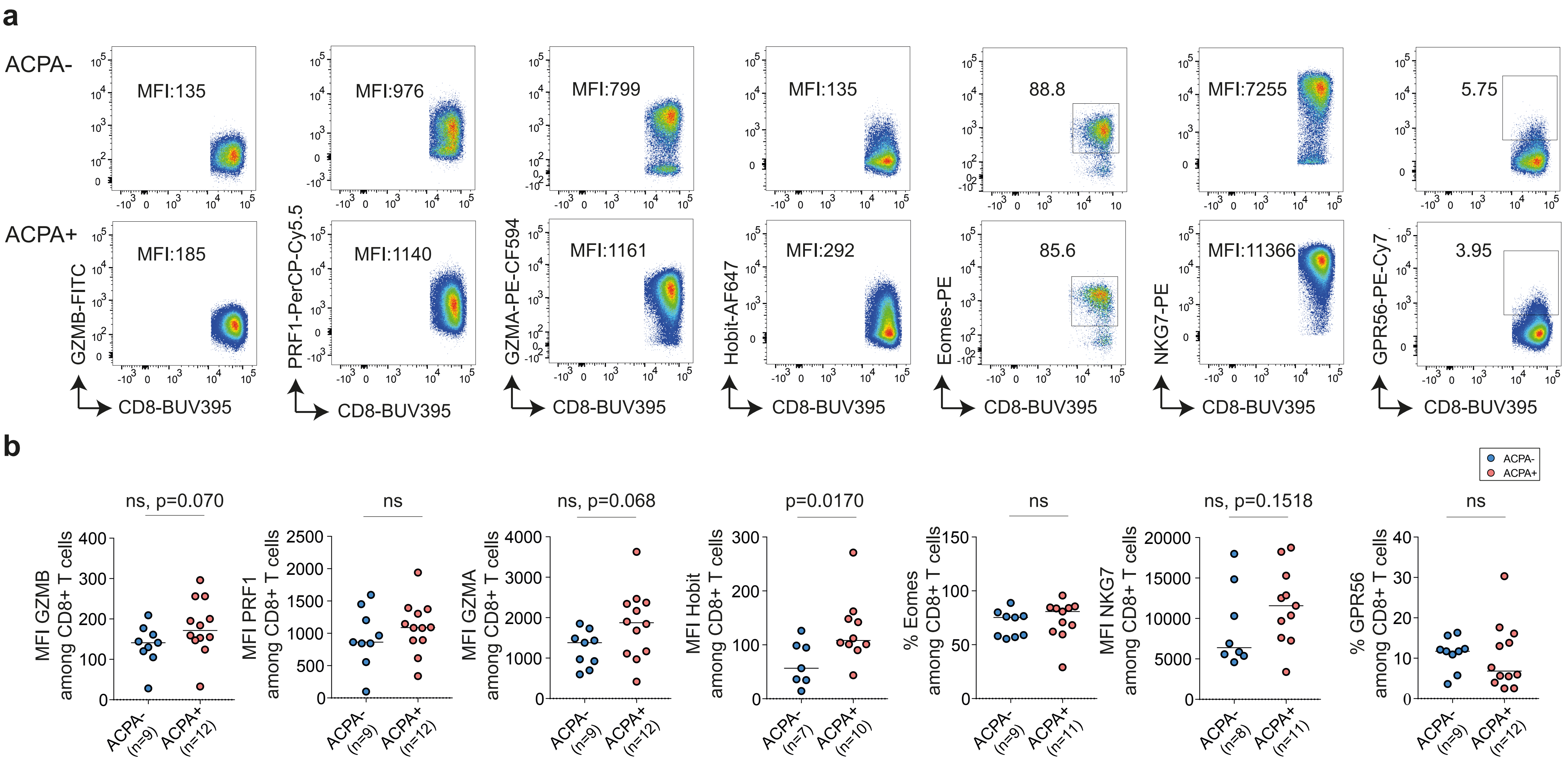

Supplementary Figure 2. Cytotoxic markers in CD8+ T cells in SF of RA patients. a) Representative flow cytometry dot plot staining of effector molecules, receptors and transcription factors associated with cytotoxic functions in CD8+ T cells from synovial fluid (SF) from ACPA- (upper panel) and ACPA+ (lower panel) RA patients quantified in **b)**, (ACPA-, n=7-9) (ACPA+, n=10-12). Line represents median, two-tailed Mann-Whitney U test, ns: not significant. Data are from a pool of nine independent experiments where a circle is a single replicate. Blue dots indicate ACPA- RA SF and red dots indicate ACPA+ RA SF.

Supplementary Figure 3

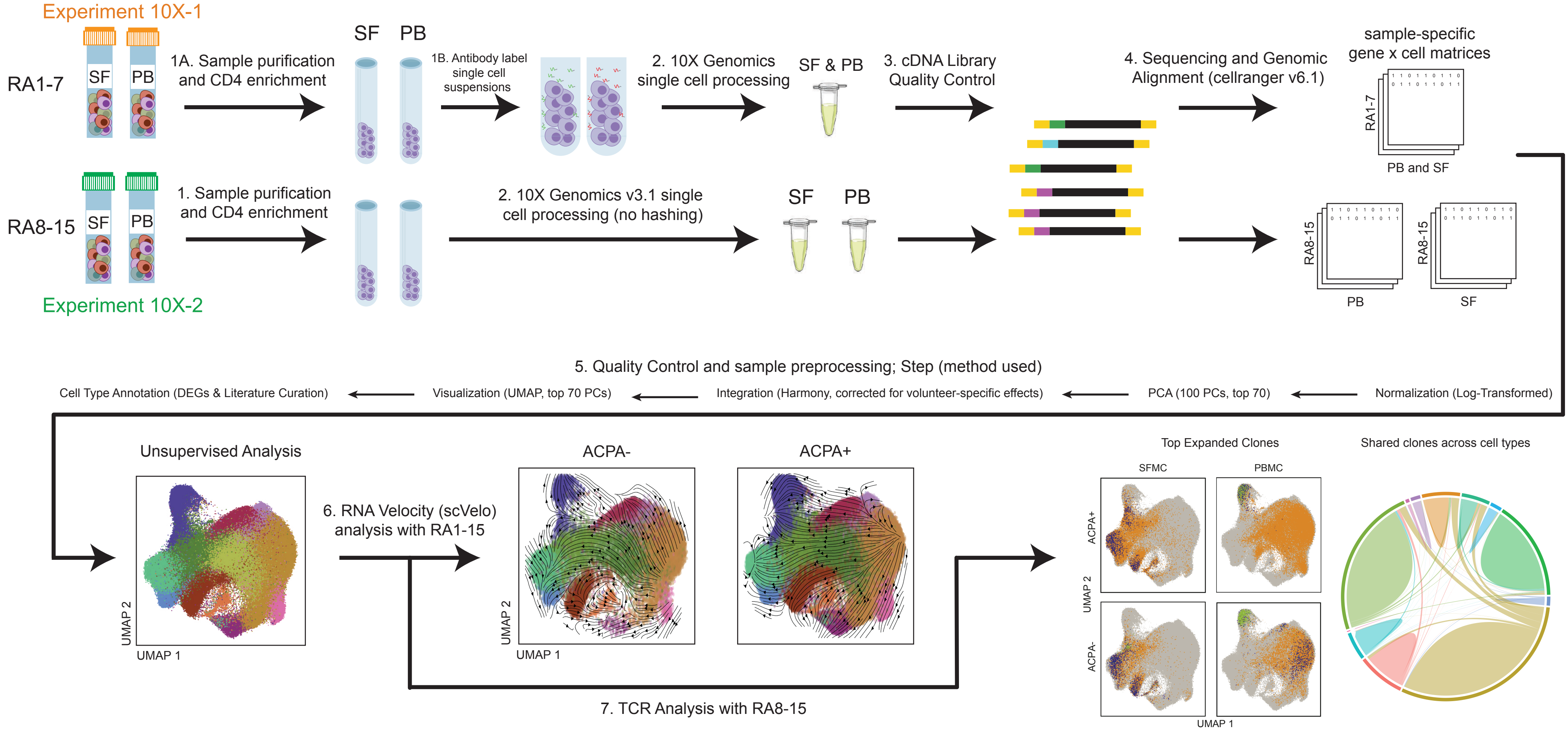

Supplementary Figure 3. Overview of Single Cell RNA-Sequencing Pipeline. Cryopreserved PBMC and SFMC samples were first thawed and washed before enriching for CD4+ T Cells (Step 1/1A). Once enriched, RA1-7 were labeled with antibodies (hashing) for sample multiplexing and downstream single cell capture using the 10X Chromium controller (1B, 2). RA8-15 PBMC and SFMCs samples were prepared as those from RA1-7 except samples were processed independently for single cell capture (i.e., samples were not multiplexed using antibodies, 2). Following cell capture and reverse transcription, the stable cDNA product underwent amplification, library preparation, and standard quality control (QC) to ensure libraries could proceed with sequencing (3). Libraries that passed QC metrics were then sequenced on a NextSeq500/550 v2 kit and aligned using the 10X cellranger pipeline (v6.1; 4). Sample-specific gene x cell matrices underwent QC and preprocessing using a standard single cell computational workflow (5). All samples (i.e., RA1-15) were used in the unsupervised analysis and cell annotations, as well as the RNA velocity analysis (6). Because RA8-15 samples retained compartment specificity, they were subset and used for the TCR clonality analysis (7).

Supplementary Figure 4

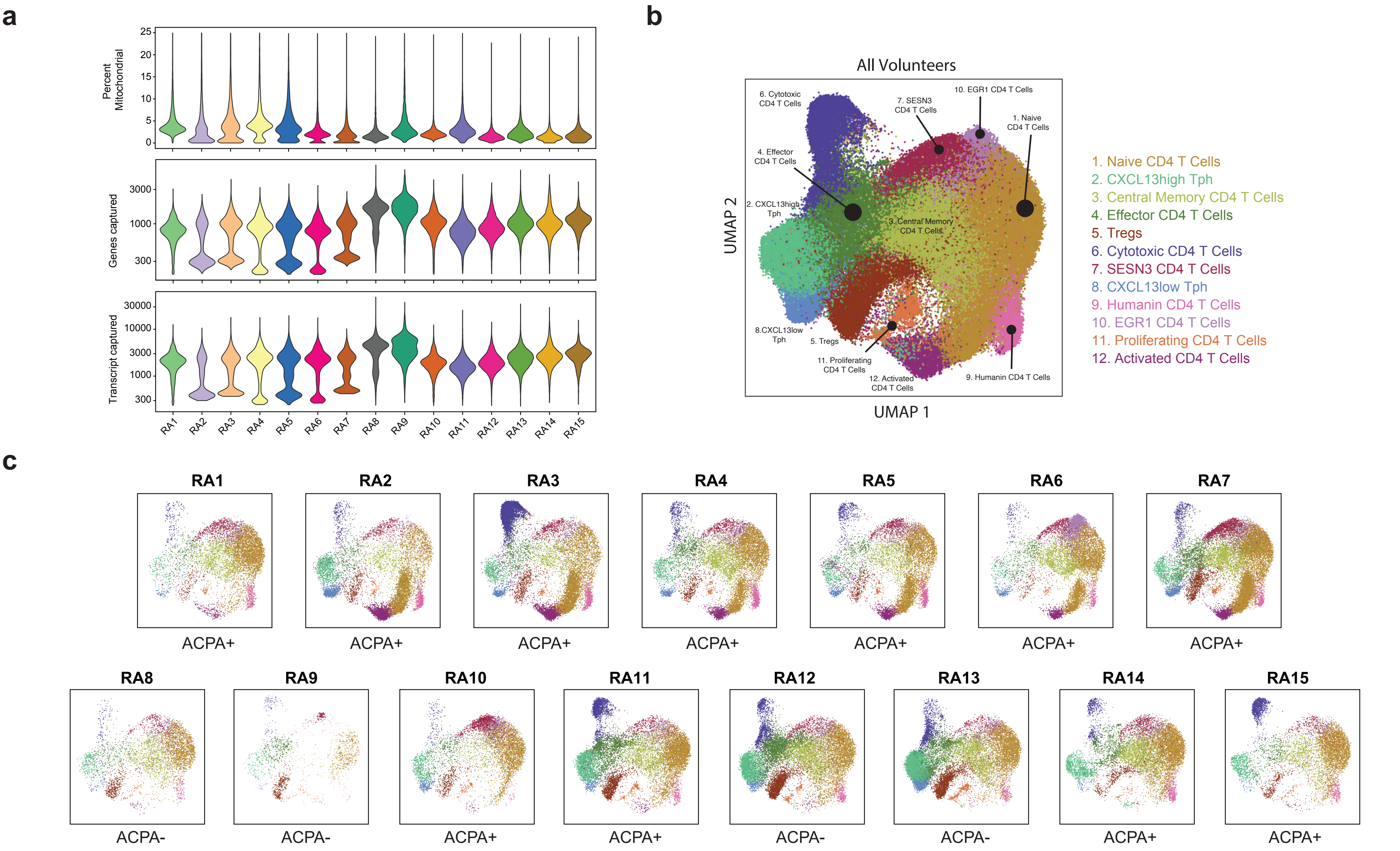

Supplementary Figure 4. Single cell RNA sequencing of CD4+ T cells from RA patients. a) Violin plots showing the frequency of mitochondrial genes (upper panel), the number of genes captured (middle panel) and the total number of transcripts (lower panel) per patient. **b**) UMAP plot of 166,944 cells colored by cell type and **c**) split by patients

Supplementary Figure 5

a

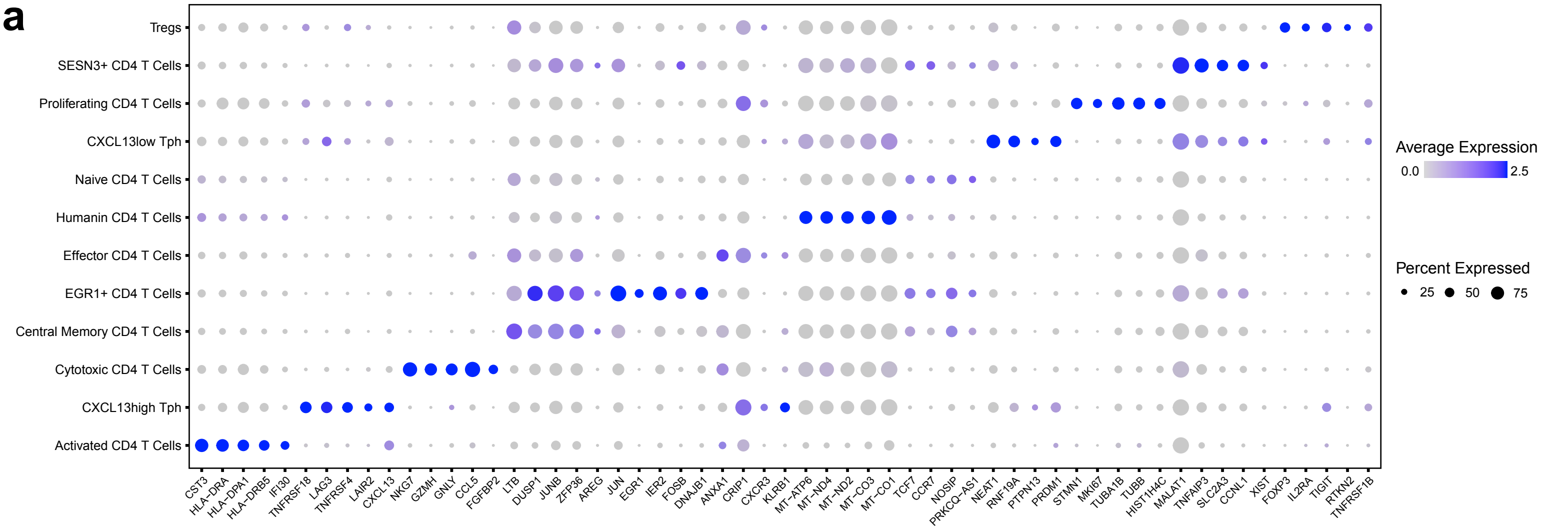

b

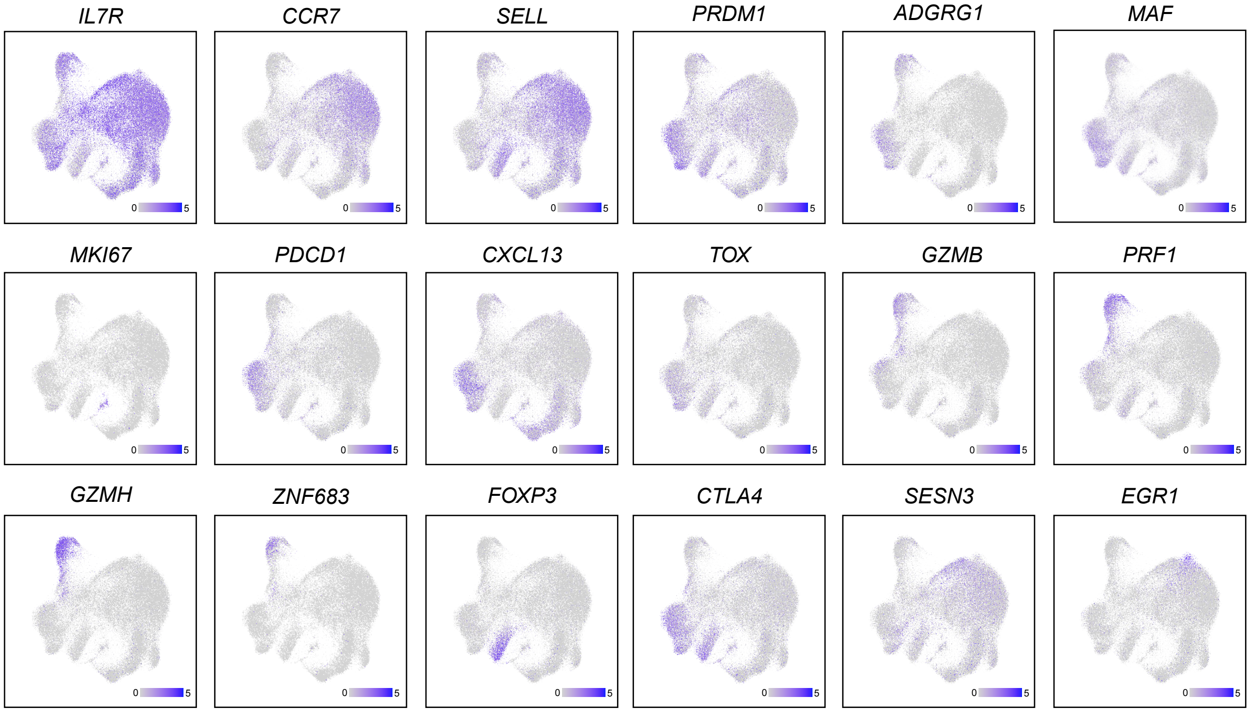

c

Cluster number	Cluster Name	Azymuth cell types 2021	Common DEG	Adjusted p-value
1	Naive CD4 T cells	CD4+ Naive T CL0000895	*PIK3IP1,FHIT,NUCB2,NOSIP,CCR7,TCF7,LEF1, LDHB,MAL*	2.859e-13
3	Central Memory CD4 T cells	CD4+ Central Memory T3 CL0000897	*LTB,IL7R,AQP3,MAL,LDHB*	1.926e-8
4	Effector CD4 T cells	CD4+ Effector Memory T1 CL0001044	*ITGB1,IL7R,CCL5,KLRB1,GZMA,DUSP2*	1.525e-10
6	Cytotoxic CD4 T cells	CD4+ Cytotoxic T CL0000934	*NKG7,GNLY,FGFBP2,CST7,CCL5,ITGB1, IL32,GZMA,B2M,GZMH,S100A4*	5.587e-19
11	Proliferating CD4 T cells	CD4+ Proliferating T CL0000896	*CENPF,PTTG1,MKI67,TOP2A,TPX2, BIRC5,NUSAP1,ASPM,TYMS,PCLAF,RRM2*	2.108e-17
5	Tregs	Memory Regulatory T CL0000815	*S100A4,B2M,IZKF2,TIGIT,CTLA4,FOXP3,RTKN2, IL2RA*	8.580e-9
7	SESN3	CD4+ Memory/Effector T CL0000624	*MALAT1, ITK, CD96, PDCD4, PTPRC*	5.568e-7
10	EGR1	CD4+ Naive T CL0000895	*PIK3IP1, MAL, LEF1, LDHB, TCF7, NOSIP, CCR7*	1.623-9

Cluster number	Cluster Name	BioPlanet 2019	Common DEG	Adjusted p-value
7	SESN3	Interleukin-2 signaling pathway	*JAK1, SOC3, STAT3, JAK3, SFPQ, FOS, PIK3R1, JUN, CDKN1B, IL6ST, DUSP1, SELL, ABLIM1*	3.401e-26
		**MSigDB Hallmark 2020**	**Common DEG**	**Adjusted p-value**
		Interferon Gamma Response	*IRF1, BTG1, NFKB1, CD69, TNFAIP3, IL10RA, PDE4B, HIF1A, SOCS3*	5.587e-19
		TNF-alpha Signaling via NF-kB	*IRF1, JUN, BTG1, KLF6,NFKB1, CD69, CD44, BTG2, FOS, SLC2A3, TNFAIP3, IL6ST, PDE4B, DUSP1, SOCS3, ATP2B1, ZFP36, CDKN1B, IL10RA, HIF1A*	5.552e-17
		**NCI-Nature 2016**	**Common DEG**	**Adjusted p-value**
		IL6-mediated signaling events	*JUN, FOS, PIK3R1, STAT3, JAK1, IRF1, JUNB, SOCS3, PIAS1*	2.622e-7

Supplementary Figure 5. Top differentially expressed genes (DEGs) in CD4+ T cell clusters from 4 ACPA- and 11 ACPA+ RA patients. **a**) 2-D dot plots showing the expression of the top DE genes in each cluster (circle size indicates percentage of cells expressing, color intensity indicates average expression) **b**) UMAP feature plots showing key genes used for cluster annotation. **c**) Cluster type cross-reference with Azimuth 2021 cell types from EnrichR (Left panel) and Cluster 7 comparison with EnrichR databases (Right panel), Fisher exact test.

Supplementary Figure 6

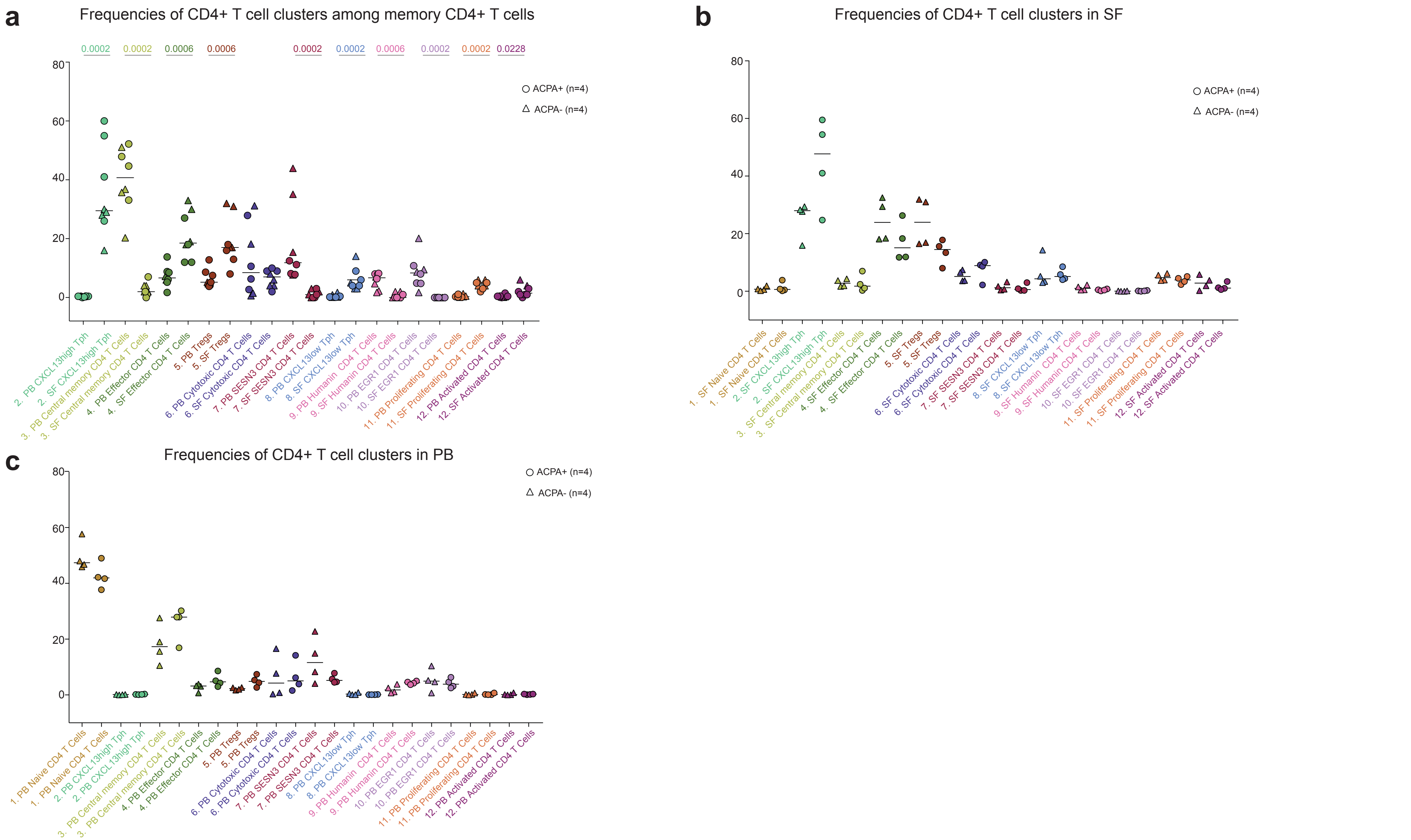

Supplementary Figure 6. CD4+ T cell clusters in ACPA+ and ACPA- RA. a) Frequencies of CD4+ T cell clusters among memory CD4+ T cells in PB and SF from n=8 RA patients (4 ACPA+, 4 ACPA-). **b**) Frequencies of CD4+ T cell clusters in SF from n=8 RA patients (4 ACPA+, 4 ACPA-). **c**) Frequencies of CD4+ T cell clusters in PB from n=8 RA patients (4 ACPA+, 4 ACPA-). Circle indicates ACPA+ patients, triangle indicates ACPA- patients. Line represents median, two-tailed Mann-Whitney U test.

Supplementary Figure 7

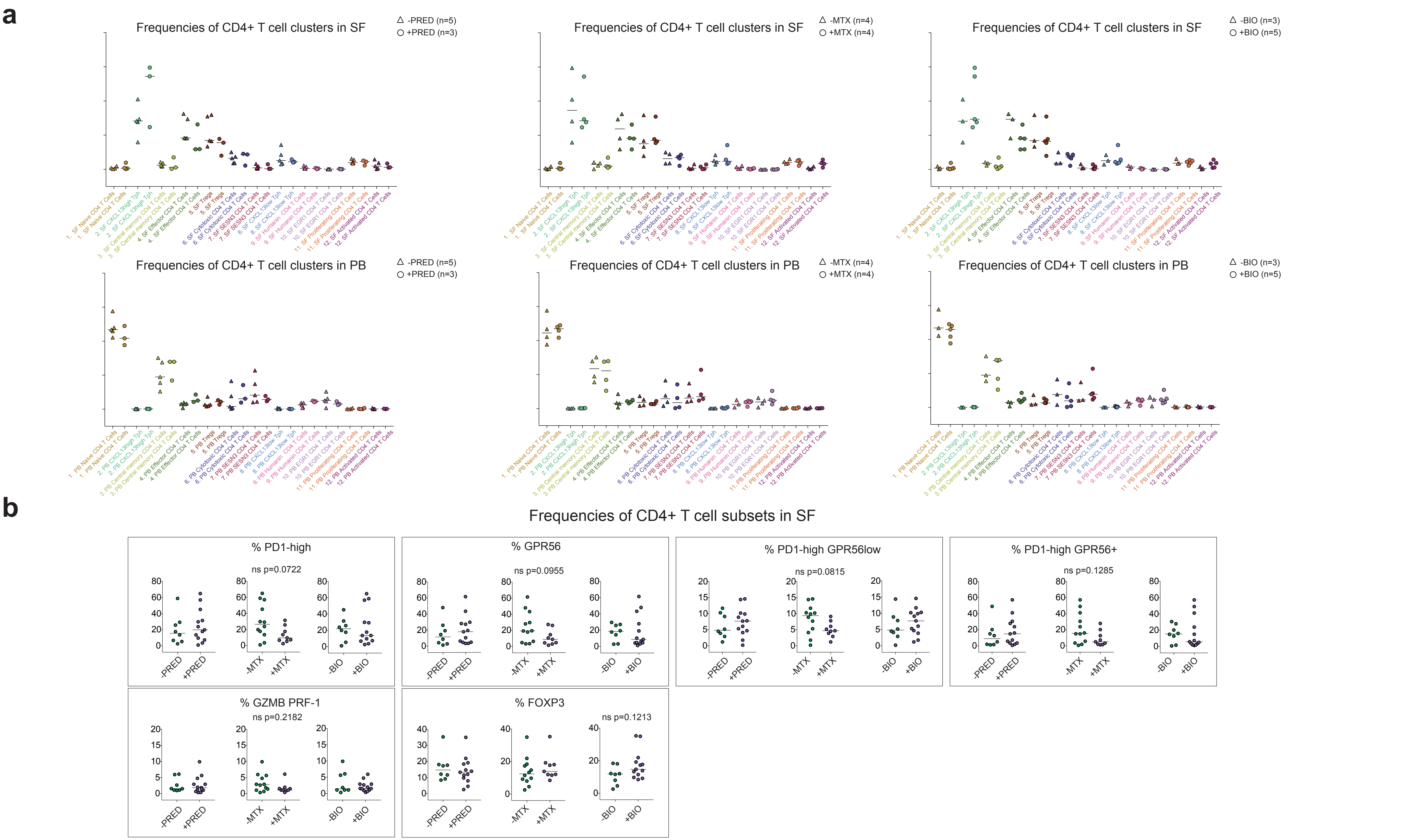

Supplementary Figure 7. Frequencies of CD4+ T cells in patients undergoing treatment at time fo sampling. a) Frequencies of the different CD4+ T cell clusters in SF (upper panels) and PB (lower panels) in patients undergoing (circle) or not (triangle) prednisolone (PRED) (-PRED n=5, +PRED n=3, left panels), methotrexate (MTX) (MTX n=4, +MTX n=4, middle panels) or biologics (BIO) (-BIO n=3, +BIO n=5, right panels) at time of sampling. **b)** Frequency of PD1-high, GPR56+, PD1-high GPR56low, PD1-high GPR56+,GZMB PRF-1 and FOXP3 in SF of patients undergoing (purple dot) or not (green dot) prednisolone (PRED) (-PRED n=8, +PRED n=13), methotrexate (MTX) (-MTX n=12, +MTX n=9) or biologics (BIO) (-BIO n=8, +BIO n=13) at time of sampling. Line represents median, two-tailed Mann-Whitney U test, ns: not significant.

Supplementary Figure 8

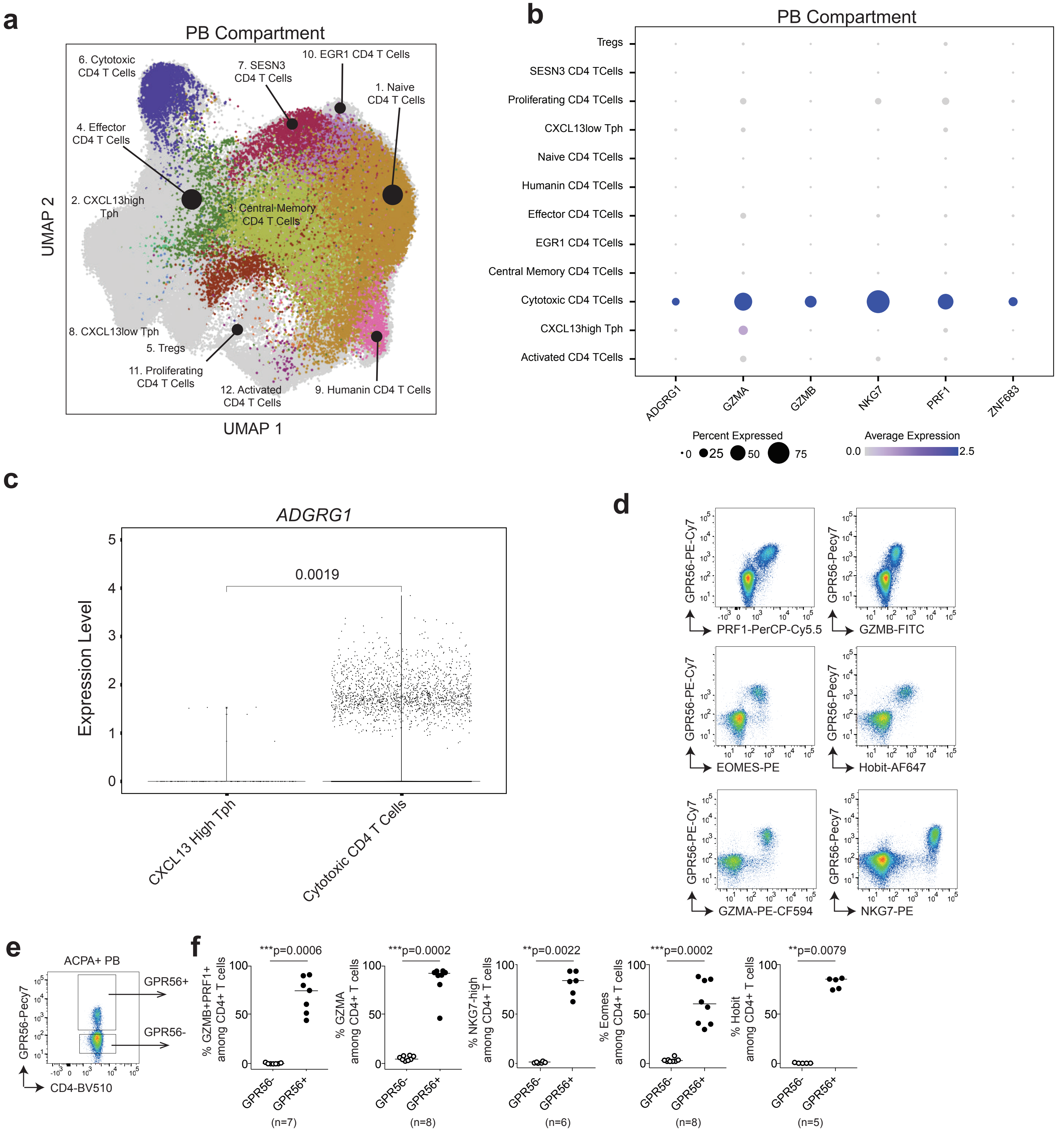

Supplementary Figure 8. Cytotoxic CD4+ T cells and *ADGRG1*/GPR56 expression in PB from ACPA+ RA patients. **a**) UMAP displaying 12 CD4+ T cell clusters in PB in 4 ACPA+ and 4 ACPA- RA patients. **b**) 2-D dot plots showing the expression of selected genes in the different CD4+ T cell clusters in RA PB (circle size indicates percent expressed, color intensity indicates average expression). **c**) *ADGRG1* expression in CXCL13high TPH and cytotoxic CD4+ T cell clusters in PB (n=4 ACPA+ RA), two-tailed Mann-Whitney U test. **d**) Representative flow cytometry dot plots showing the expression of effector molecules, receptors and transcription factors associated with cytotoxic functions in CD4+ T cells in ACPA+ RA. **e**) Representative flow cytometry dot plots showing GPR56 expression among CD4+ T cells in ACPA+ RA PB. **f**) Frequency of GZMB+ PRF-1+ (n=7, p=0.0006), GZMA (n=8, p=0.0002), NKG7-high (n=6, p=0.0022), Eomes (n=8, p=0.0002), Hobit (n=5, p=0.0079) in GPR56-negative and positive CD4+ T cells in ACPA+ RA PB. Line represents median, two-tailed Mann-Whitney U test. Data are from a pool of seven independent experiments where a circle is a single replicate.White dots indicate GPR56- CD4+ T cells and black dots indicate GPR56+ CD4+ T cells.

Supplementary Figure 9

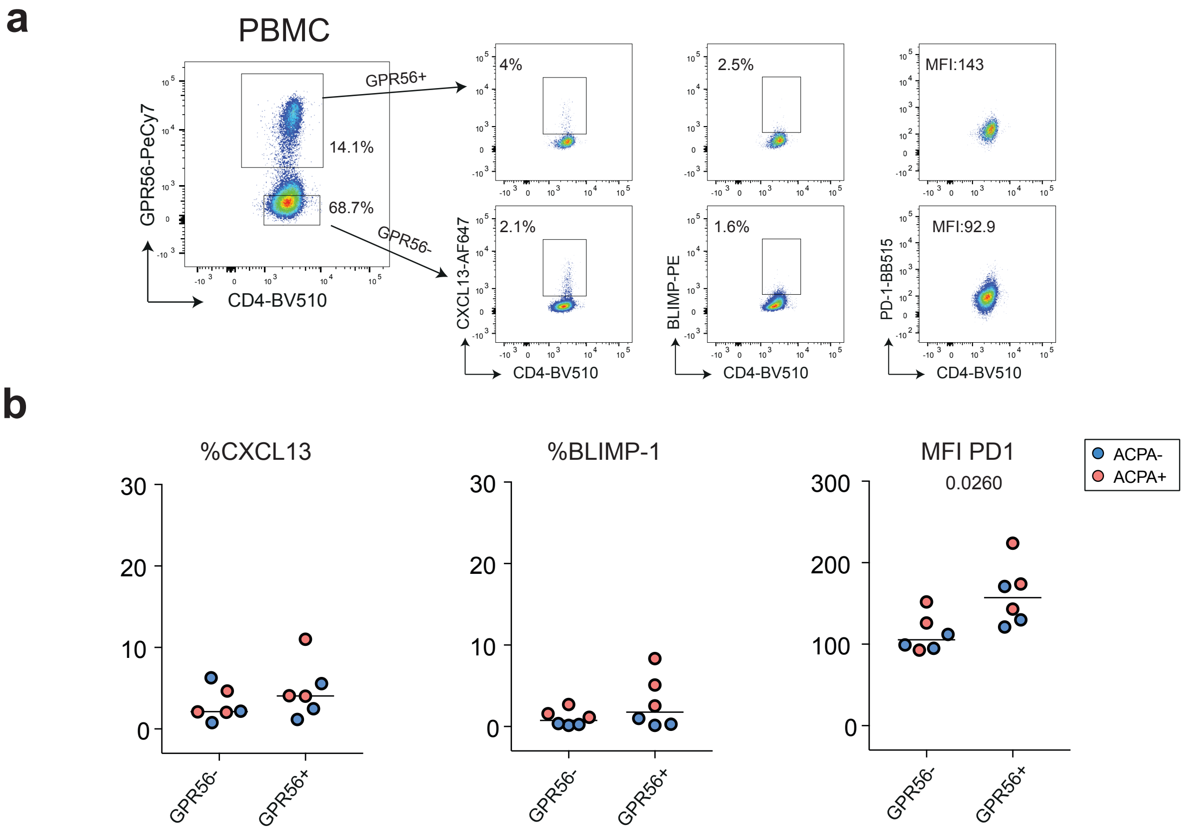

Supplementary Figure 9. CXCL13 expression in GPR56+ CD4+ T cells. a) Representative flow cytometry dot plot showing the expression of CXCL13, BLIMP-1 and PD-1 within GPR56- and GPR56+ CD4+ T cells after 3 hours of CD3/CD28 activation in PBMC from a RA patient quantified in **b**), n=6 RA patients. Data are from a pool of six independent experiments where a circle is a single replicate. Blue dots indicate ACPA- RA and red dots indicate ACPA+ RA patients. Two-tailed Mann-Whitney U test.

Supplementary Figure 10

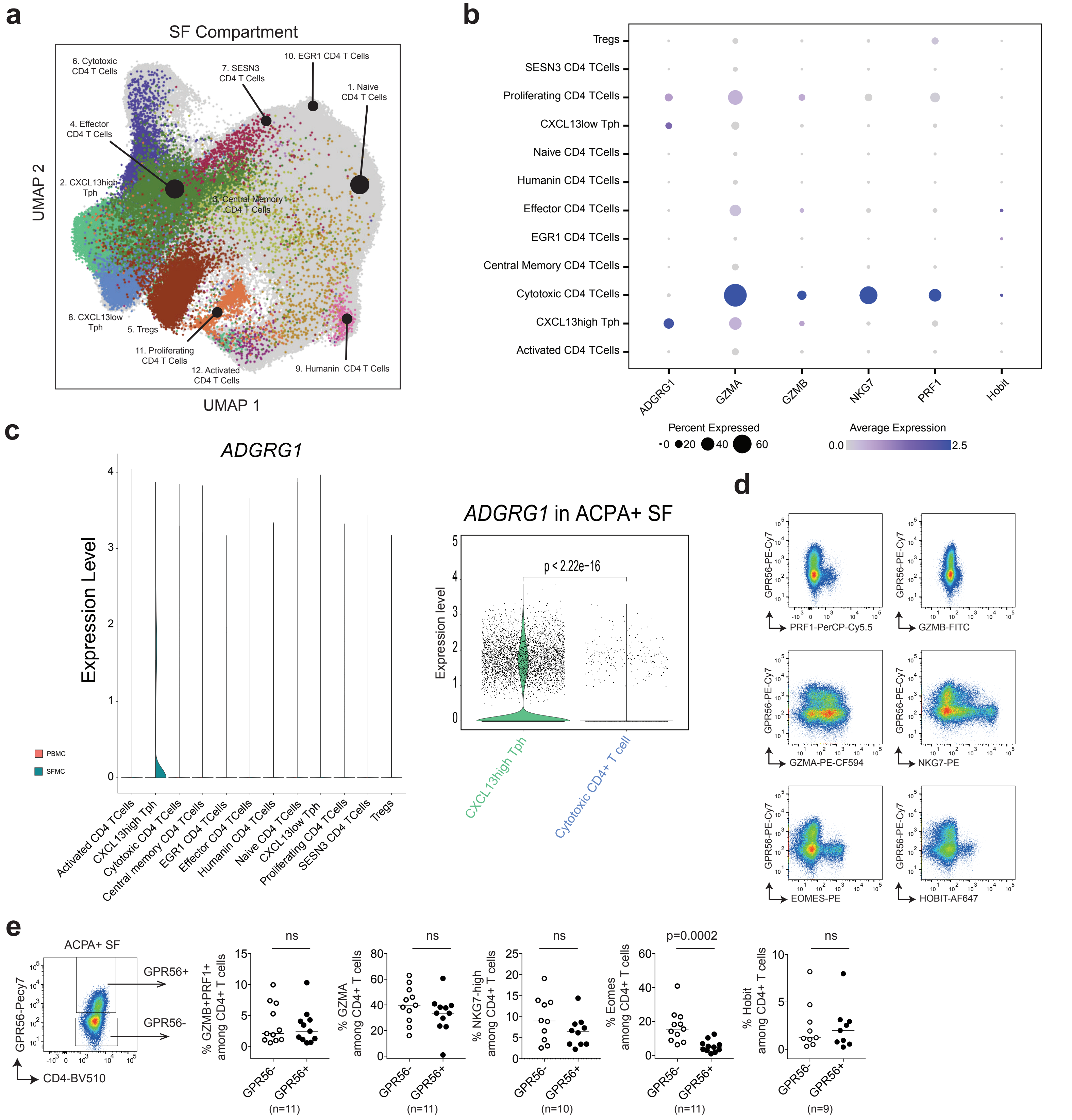

Supplementary Figure 10. Cytotoxic CD4+ T cells and *ADGRG1*/GPR56 expression in SF from RA patients.a) UMAP displaying 12 CD4+ T cell clusters in SF in 8 RA patients (4 ACPA+, 4 ACPA-). **b**) 2-D dot plots showing the gene expression of selected genes encoding cytotoxic effector molecules and transcription factors in SF in 8 RA patients (4 ACPA+, 4 ACPA-). **c**) *ADGRG1* expression in PB and SF CD4+ T cells clusters (n=4 ACPA+) (left panel) and in CXCL13high TPH versus cytotoxic CD4+ T cells in ACPA+ SF (n=4) (right panel), two-tailed Mann-Whitney U test. **d**) Representative flow cytometry dot plots showing the expression of effector molecules, receptors and transcription factors associated with cytotoxic functions in CD4+ T cells in ACPA+ RA SF **e**) Frequency of GZMB+PRF1+ (n=11), GZMA (n=11), NKG7-high (n=10), Eomes (n=11, p=0.0002) and Hobit in GPR56-negative and positive CD4+ T cells in ACPA+RA SF. Line represents median, two-tailed Mann-Whitney U test, ns: not significant. Data are from a pool of nine independent experiments where a circle is a single replicate. White dots indicate GPR56- CD4+ T cells and black dots indicate GPR56+ CD4+ T cells.

Supplementary Figure 11

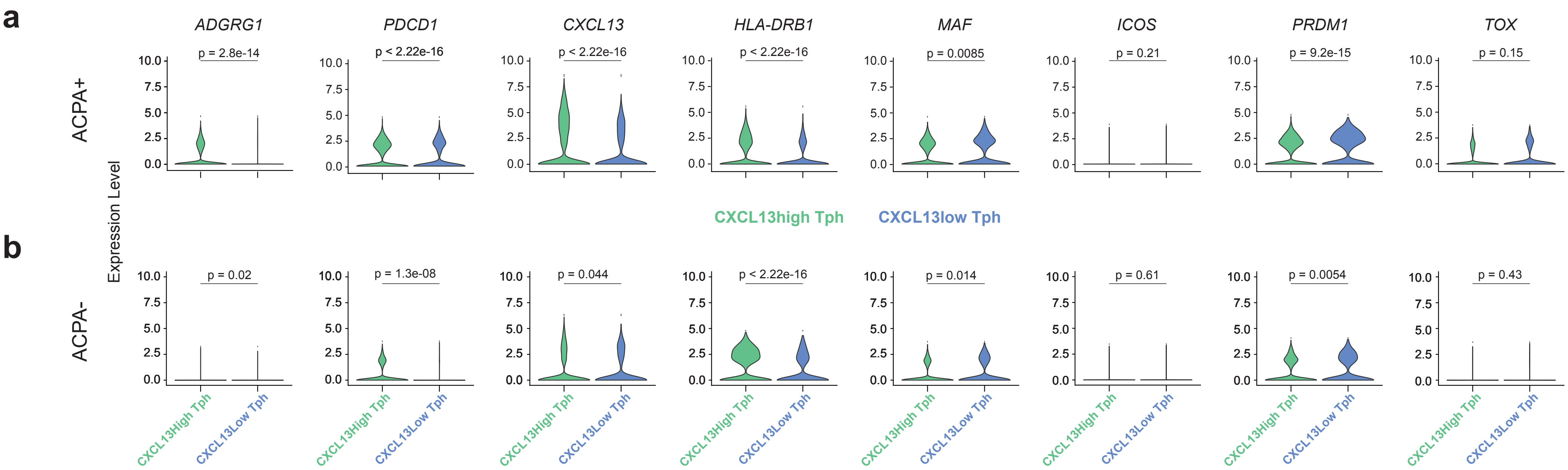

Supplementary Figure 11. Violin plots displaying the gene expression levels of TPH-associated genes in CXCL13high and CXCL13low TPH cells in ACPA+ (**a**) and ACPA- RA SF (**b**). (n=4 ACPA- SF and 4 ACPA+ SF). Line represents median, two-tailed Mann-Whitney U test

Supplementary Figure 12

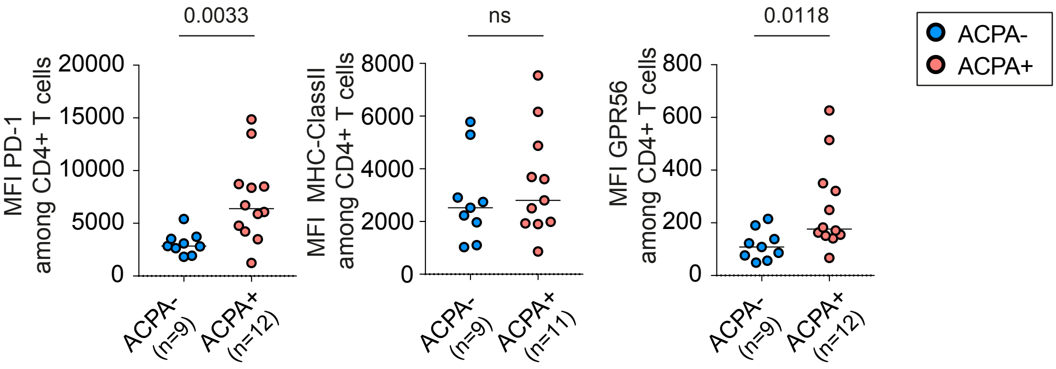

Supplementary Figure 12. PD1, MHCII and GPR56 expression on CD4+ T cells in ACPA+ and ACPA- RA SF. PD1, MHCII and GPR56 MFI (mean fluoresecence intensity) on CD4+ T cells in ACPA- and ACPA+ RA SF (n=9 ACPA- SF and n=11-12 ACPA+ SF). Data are from a pool of nine independent experiments where a circle is a single replicate.median, two-tailed Mann-Whitney U test. ns: not significant. Blue dots indicate ACPA- RA SF and red dots indicate ACPA+ RA SF.

Supplementary Figure 13

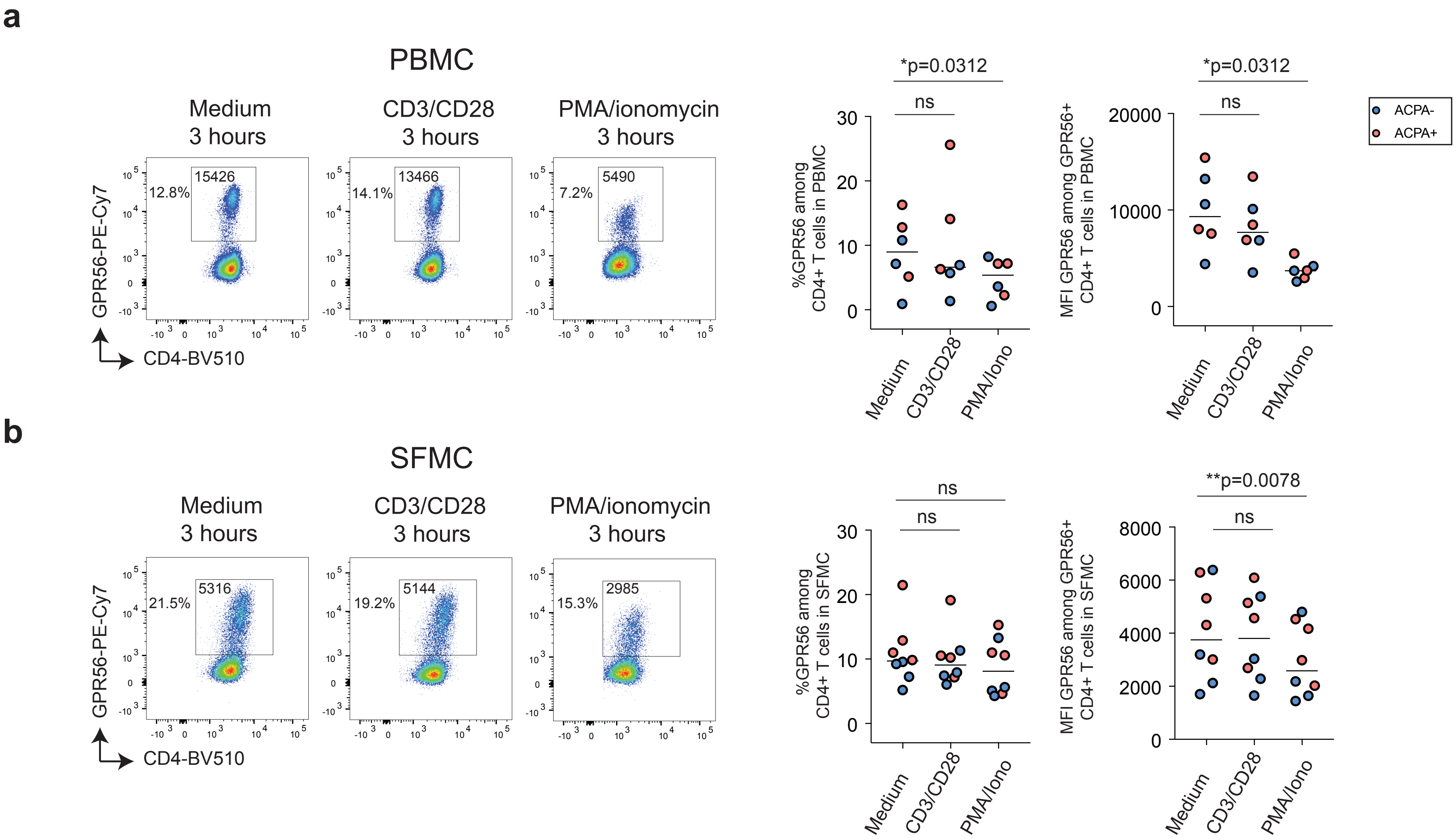

Supplementary Figure 13. GPR56 downregulation after activation. **a**) Representative flow cytometry staining showing GPR56 expression (% and mean fluorescence intensity) on PB CD4+ T cells after 3 hours activation with CD3/CD28 beads or PMA/Ionomycin and quantified in 6 RA patients (n=3 ACPA-, n=3 ACPA+). Data are from a pool of 3 independent experiments where a circle is a single replicate.**b**) Representative flow cytometry staining showing GPR56 expression on SF CD4+ T cells after 3 hours activation with CD3/CD28 beads or PMA/Ionomycin and quantified in 8 RA patients (n=4 ACPA-, n=4 ACPA+). Data are from a pool of 4 independent experiments where a circle is a single replicate.Line indicates median, two-tailed Wilcoxon paired test. ns: not significant. Blue dots indicate ACPA- and red dots indicate ACPA+ RA patients.

Supplementary Figure 14

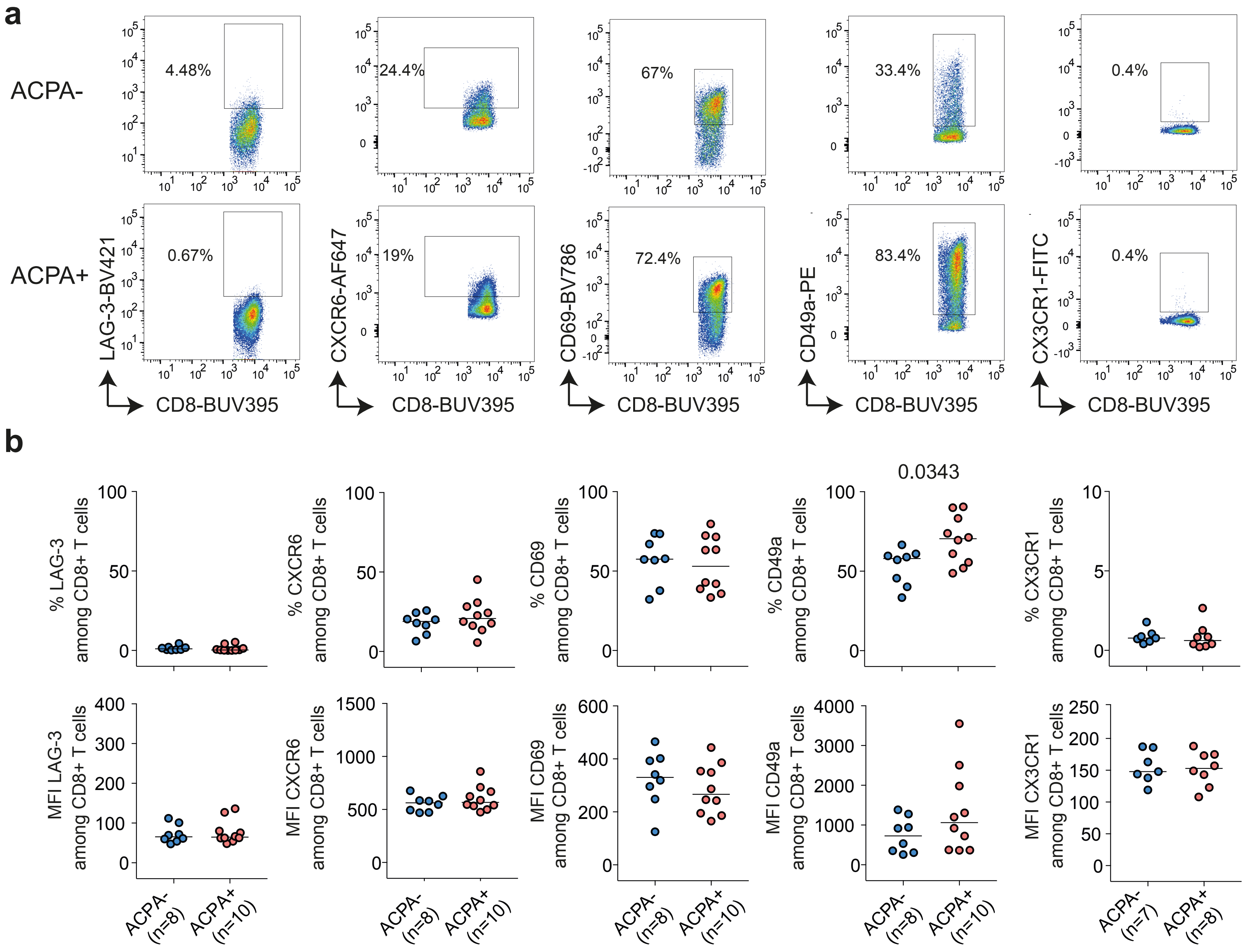

Supplementary Figure 14. Tissue-resident memory receptors on CD8+ T cells in SF from RA patients. a) Representative flow cytometry dot plots showing the expression of LAG-3, CXCR6, CD69, and CX3CR1 in ACPA- and ACPA+ SF CD8+ T cells, quantified in **b)** LAG-3, CXCR6, CD69, CD49a (p=0.0343) (ACPA- n=8; ACPA+ n=10).and CX3CR1 (ACPA- n=7; ACPA+ n=8). Line represents median, two-tailed Mann-Whitney U test. Data are from a pool of eight independent experiments where a circle is a single replicate. Blue dots indicate ACPA- RA SF and red dots indicate ACPA+ RA SF.
