# Supplementary material for: Single cell sequencing identifies clonally expanded synovial CD4+ TPH cells expressing GPR56 in rheumatoid arthritis
Source: Nat Commun. 2022 Jul 13;13:4046. doi: 10.1038/s41467-022-31519-6 (PMC9279430; doi:10.1038/s41467-022-31519-6)
Supplement: Supplementary file 3 — Description of Additional Supplementary Files [file 41467_2022_31519_MOESM3_ESM.pdf]

Description of Additional Supplementary Files

Supplementary Data 1. Patient characteristics

Supplementary Data 2. $CD4^+$ T cell type composition in PBMC and SFMC in RA patients (n=4 ACPA-, n=4 ACPA+) (Frequencies)

Supplementary Data 3. Differentially expressed genes in the 12 $CD4^+$ T cell clusters in combined peripheral blood and synovial fluid (n=15 RA patients, 11 ACPA+, 4 ACPA-)

Supplementary Data 4. CDR3 alpha/beta amino-acid sequences sequences in annotated $CD4^+$ T cell clusters in peripheral blood in n=8 RA patients (4 ACPA- and 4 ACPA+)

Supplementary Data 5. CDR3 alpha/beta amino-acid sequences sequences in annotated $CD4^+$ T cell clusters in synovial fluid in n=8 RA patients (4 ACPA- and 4 ACPA+)

Supplementary Data 6. Phenotype ($CD4^+$ T cell clusters) and TCR clonality sharing of expanded $CD4^+$ T cell clones

a. TCR a/b recovery in $CD4^+$ T cells in SF and PB (n=4 ACPA+; n=4 ACPA-)
b. Phenotype of expanded $CD4^+$ T cell clones in ACPA+ SF (n=4) and ACPA- (n=3)
c. Phenotype of expanded $CD4^+$ T cell clones in ACPA+ PB (n=4) and ACPA- (n=3)
d. Phenotype of expanded $CD4^+$ T cell clones which are shared between $CD4^+$ T cell clusters (n=4 ACPA+ SF and n=3 ACPA- SF)
e. Sharing of CDR3s with CXCL13high T_{PH} (first table), Tregs (second table), effector $CD4^+$ T cells (third table) and cytotoxic $CD4^+$ T cells (fourth table) clones in ACPA+ SF (n=4) and ACPA- (n=3)

Supplementary Data 7. CDR3 alpha/beta amino acid sequences shared in annotated $CD4^+$ T cell clusters in synovial fluid in ACPA+ SF (n=4) and ACPA- (n=3)

Supplementary Data 8. Flow cytometry antibodies

Supplementary Data 9. Flow cytometry panels
